# Population-Tailored Care for Homeless Veterans and Acute Care Use, Cost, and Satisfaction: A Prospective Quasi-Experimental Trial

**DOI:** 10.5888/pcd15.170311

**Published:** 2018-02-15

**Authors:** Thomas P. O’Toole, Erin E. Johnson, Matthew Borgia, Amy Noack, Jean Yoon, Elizabeth Gehlert, Jeanie Lo

**Affiliations:** 1 National Center on Homelessness Among Veterans, US Veterans Health Administration, Providence, Rhode Island; 2 Providence VA Medical Center, Providence, Rhode Island; 3 Alpert Medical School at Brown University, Providence, Rhode Island; 4 San Francisco VA Medical Center, San Francisco, California; 5 University of California, San Francisco, San Francisco, California; 6 VA Palo Alto Health Care System, Palo Alto, California

## Abstract

**Introduction:**

Although traditional patient-centered medical homes (PCMHs) are effective for patients with complex needs, it is unclear whether homeless-tailored PCMHs work better for homeless veterans. We examined the impact of enrollment in a Veterans Health Administration (VHA) homeless-tailored PCMH on health services use, cost, and satisfaction compared with enrollment in a traditional, nontailored PCMH.

**Methods:**

We conducted a prospective, multicenter, quasi-experimental, single-blinded study at 2 VHA medical centers to assess health services use, cost, and satisfaction during 12 months among 2 groups of homeless veterans: 1) veterans receiving VHA homeless-tailored primary care (Homeless-Patient Aligned Care Team [H-PACT]) and 2) veterans receiving traditional primary care services (PACT). A cohort of 266 homeless veterans enrolled from June 2012 through January 2014.

**Results:**

Compared with PACT patients, H-PACT patients had more social work visits (4.6 vs 2.7 visits) and fewer emergency department (ED) visits for ambulatory care-sensitive conditions (0 vs 0.2 visits); a significantly smaller percentage of veterans in H-PACT were hospitalized (23.1% vs 35.4%) or had mental health–related ED visits (34.1% vs 47.6%). We found significant differences in primary care provider–specific visits (H-PACT, 5.1 vs PACT, 3.6 visits), mental health care visits (H-PACT, 8.8 vs PACT, 13.4 visits), 30-day prescription drug fills (H-PACT, 40.5 vs PACT, 58.8 fills), and use of group therapy (H-PACT, 40.1% vs PACT, 53.7%). Annual costs per patient were significantly higher in the PACT group than the H-PACT group ($37,415 vs $28,036). In logistic regression model of acute care use, assignment to the H-PACT model was protective as was rating health “good” or better.

**Conclusion:**

Homeless veterans enrolled in the population-tailored primary care approach used less acute care and costs were lower. Tailored-care models have implications for care coordination in the US Department of Veterans Affairs VA and community health systems.

## Introduction

Heath care for socially disadvantaged, high-risk, or high-need patients such as homeless persons is often defined by high rates of acute care service use and poor clinical outcomes (1–7). Optimizing health care delivery to these patients is a clinical and fiscal challenge. The development of patient-centered medical homes (PCMHs) is one approach that has received increased attention recently (8), and the approach has been adopted in health systems with generally positive results across several domains (9). This proactive care team approach centers on patients, enhances care management and coordination, and is particularly effective for patients with complex care needs (10,11). However, we know little about how this model may work in vulnerable and homeless populations, where competing social needs, environmental conditions, and social determinants of health make acute care and chronic disease management more difficult.

There is a substantial commitment to providing care for homeless persons through Health Care for the Homeless clinics based in federally qualified health centers and shelters (12), in the Veterans Health Administration (VHA), and through voluntary and charitable efforts (13). These homeless population–tailored models are typically distinguished by locations convenient to homeless persons, easier access and scheduling processes, integrated social services, cultural sensitivity, and enhanced case management. Some studies showed that homeless patients had higher satisfaction levels when health care was provided in a population-tailored setting (14,15). Another study found that a population-tailored approach was more effective than a nontailored approach in engaging homeless persons who were new to care (16) and in reducing the use of acute care services, albeit often after a substantial lag period. However, other studies of health care use among homeless persons found that being assigned to primary care was associated with being hospitalized and having frequent readmissions (17,18). This paradox may reflect increases in feelings of self-efficacy among homeless persons: they may feel empowered to seek care when their health conditions are identified in primary care settings, but the health conditions cannot be satisfactorily addressed in these settings. Alternatively, the paradox may reflect the inability of traditionally organized primary care to engage homeless persons in the spectrum of care and services in an on-demand capacity. Rigorous analyses of primary care models tailored for the homeless population are typically limited to intragroup pre–post comparisons, case series, secondary analyses, or studies that use historic controls for comparison (19–22). Thus, it is difficult to assess how various health care models affect the use of health care services by homeless persons.

The objective of this study was to compare health care service use and cost outcomes among homeless veterans enrolled in a traditional (not tailored to a homeless population) PCMH with outcomes among homeless veterans enrolled in a homeless population–tailored PCMH. Our hypothesis was that a population-tailored model would be associated with less use of acute care services because 1) greater use of ambulatory care services would result from enhanced trust and facilitated access to such services and/or 2) underlying social determinants of health would be more effectively addressed in the population-tailored model. We examined use of VHA and community-based primary care, specialty care, mental health care, and acute care services by homeless veterans during a 12-month period. Findings from our study may be generalizable to other high-risk patient populations with unique care needs that are coupled with challenges engaging in and accessing care in traditional settings.

## Methods

This was a prospective, multicenter, quasi-experimental, single-blinded trial that compared patterns of health care service use and cost among homeless veterans enrolled in care at VHA facilities in San Francisco, California, and Providence, Rhode Island. Data collection took place from June 2012 through January 2014. VHA Central Institutional Review Board approval was obtained for this study (C-IRB Study no. 11–06).

**Study population.** The study population consisted of homeless veterans who were enrolled in one of 2 VHA-based PCMH models. PACT (Patient Aligned Care Team) is the traditional PCMH model available to all VHA-eligible veterans. H-PACT (Homeless-PACT) is the homeless-tailored PCMH model. Study sites that enrolled veterans in this study operate both forms of PCMH. Veterans had already been assigned to their PCMH model by choice or by method of referral, independent of the study. Homelessness was defined according to criteria of the McKinney–Vento Act (23) following a sheltering typology that includes unsheltered (eg, living outdoors, in a car, or an abandoned building), staying in an emergency shelter, or staying in transitional housing. We also included veterans in unstable (nonpermanent) doubled-up arrangements with family or friends. Veterans who had moved into Section 8/Housing and Urban Development–Veterans Affairs Supportive Housing units were not included because this type of housing is considered permanent supportive housing. Additionally, veterans enrolled in intensive case-management programs (typically through mental health services for a persistent mental illness) at the time of study enrollment were excluded because their inclusion might have distorted the true effect of the intervention.

**Participant recruitment (June 2012–January 2014).** Patients were consented, screened for eligibility, and enrolled after referral from homeless program staff members at one of the 2 participating medical centers or after visiting the study office in response to widely posted fliers advertising the study. Patients had to be enrolled already in either of the 2 PCMH models; this enrollment was used as the basis for the quasi-experimental study arm assignment. We did not randomize patients to a clinical arm because doing so could have disrupted an established relationship between the patient and his or her clinical team and potentially jeopardized chronic disease management. For the small percentage (<3%) of veterans who were not enrolled in primary care, a research assistant described both PCMH models and how to access them for an appointment. Potential participants were instructed to return for study enrollment after they had enrolled with one of the care teams and had been seen at least once. All study participants were given $20 after completion of enrollment and again at the end of the study. Providers were not informed that their patients were enrolled in the study so as to not influence their standard of care (single blinding).

**Usual care. **PACT is a primary care–based model constructed on the principles of patient centeredness, interdisciplinary teamwork, efficiency, comprehensive whole person–oriented longitudinal care, and active communication and coordination (24). The model has demonstrated significant reductions in acute care use, improvements in satisfaction among care teams and patients, and better chronic disease management outcomes (25–27).

**Intervention.** In 2017, the H-PACT model was available in 63 VA (US Department of Veterans Affairs) facilities. Built on the framework of PACT, it has enhancements intended to address issues of access, treatment engagement, competing priorities, and the social determinants of health that are associated with homelessness (1). The H-PACT includes 1) on-site housing assistance and incorporation of homeless program staff members into the care team; 2) open access and/or care-on-demand capacity to facilitate access; 3) specialized training of staff members on issues unique to the homeless population; 4) on-site services aimed at addressing competing social needs, such as transportation, food assistance, clothing, hygiene kits, and showers; and 5) incorporation of homeless-specific protocols and monitors into clinical care (28).

**Study sites.** We studied 4 geographically distinct clinics (2 PACT clinics and 2 H-PACT clinics) at 2 VHA facilities. One PACT was located at the San Francisco VA Medical Center and the other PACT at Providence VA Medical Center. These PACTs provided usual care to enrolled homeless veterans. The H-PACTs were at the San Francisco VA Downtown Clinic and in an outbuilding on the Providence VA Medical Center campus. All 4 clinics used their respective medical center as the primary referral site for mental health, specialty care, emergency care, and hospitalizations.

### Data collection

For each enrolled participant, we collected 12 consecutive months of data on health care use, using an intention-to-treat approach and VHA electronic medical records to capture data on use of VHA-based care. We identified use events by reviewing administrative records and then conducting a manual chart review. Care events were categorized as primary care provider (PCP) and nursing visits (ie, primary care team visits), specialty care visits, social work visits, mental health care visits (group and individual), emergency department (ED) care (including ambulatory-care–sensitive conditions), or hospitalizations (including visits for acute medical/surgical, mental health, and substance abuse). We also asked all patients about any care they received outside the VA; these sites were contacted by the study team to retrieve medical records.

We collected survey data at enrollment and study completion. Questionnaires were administered by a study team member and read to participants, who could follow along with a paper copy. A research assistant recorded the responses. At enrollment, we collected data on the following sociodemographic characteristics: age; sex; race; education (dichotomized as >12 y or ≤12 y); marital status (single, never married, divorced, or separated); available cash per month through earnings, pension, disability or other sources (dichotomized as <$500 or ≥$500); sheltering status; and number of months homeless. We also collected self-reported data on the number medical and mental health conditions (participants were given a list of common conditions and an open-ended “other” option), health status (scored on a Likert scale from 1 [excellent] to 5 [poor]), alcohol and other substance use, and whether the veteran had been injured in an altercation within the previous 6 months. At study completion, participants rated their satisfaction with care received at the PACT and H-PACT clinics; these ratings were based on a Likert scale of 1 (strongly agree) to 5 (strongly disagree). 

The baseline and follow-up questionnaires were based in part on the MOS (Medical Outcomes Study) social support survey (30), a chronic disease self-efficacy questionnaire (31), the American College of Physicians’ PCMH Practice Biopsy (29), and an assessment developed by the national H-PACT program office.

Data from the VA Medical Statistical Analysis System (MedSAS) files were used to identify VA inpatient and outpatient services for study patients. VA Managerial Cost Accounting (MCA) files were used to identify VA inpatient care, outpatient care, and pharmacy costs. Data on non-VA use and costs that were sponsored by the VA were obtained from Fee Basis files. Data on non-VA hospital stays that were not sponsored by the VA were obtained through a signed release of information by the study participant. Costs of these non-VA stays were estimated based on the length of stay and information on the diagnosis-related group was obtained from hospital bills by research staff.

### Data analyses

We used χ^2 ^tests and *t* tests in Stata version 14 (StataCorp LLC) to compare demographic characteristics and care use between PACT and H-PACT groups. Differences in care use were examined for both the number of care events per person and for the proportion of veterans in each group who used those services. We considered acute care visits in terms of all-cause ED visits and hospitalizations as well as ED visits and hospitalizations for ambulatory-care–sensitive conditions based on the Agency for Healthcare Research and Quality’s Prevention Quality Indicators (32). Total annual costs of care were calculated for each patient and included costs of VA-provided care, VA-sponsored care, and non-VA–sponsored inpatient care by using 2-sided *t* tests to accommodate the data distribution in this set. 

We tested a multivariate logistic regression model using acute care use as the dependent variable. Independent variables included team assignment, health services use (primary care, social work contact, psychiatric visits, psychologist visits, and substance abuse treatment visits), social support summary scores, self-reported health rating, and demographics consistent with our a priori study hypothesis.

## Results

Overall, 266 veterans were enrolled in the study, 183 in H-PACT and 83 in PACT. The average age was 52.1 years (standard deviation, 9.2 y); most participants were male, most were single, and slightly more than half were nonwhite (Table 1). Both groups had been homeless for a little more than 2 years at baseline, and almost half had less than $500 in available cash per month. Although we found no overall difference in sheltering type between groups at enrollment, a higher percentage of the H-PACT group was in transitional housing and emergency shelters. Similar percentages (38.6% and 37.2%) moved into permanent housing by the end of the study participation.

**Table 1:** Self-Reported Sociodemographic Characteristics and Self-Reported Health Conditions of Veterans at Enrollment in the US Department of Veterans Affairs’ PACT or H-PACT, Study on Population-Tailored Care for Homeless Veterans and Acute Care Use, Cost, and Satisfaction, June 2012–January 2014

Characteristic	No. of Respondents	Overall, No. (%) (N = 266)	H-PACT^a^, No. (%^b^) (n = 183)	PACT^c^, No. (%^b^) (n = 83)	*P* Value^d^
**Sociodemographic**					
Age, mean (SD), y	265	52.1 (9.2)	51.8 (9.1)	52.7 (9.6)	.46
Male sex	265	251 (94.7)	176 (96.7)	75 (90.4)	.04
Non-Hispanic white race	264	120 (45.5)	78 (43.1)	42 (50.6)	.45
Education >12 y	264	242 (91.7)	166 (91.2)	76 (92.7)	.69
Marital status single	265	251 (94.7)	172 (94.5)	79 (96.3)	.76
Available cash per month <$500	261	120 (46.0)	82 (46.1)	38 (45.8)	.28
No. of months homeless, mean (SD)^e^	263	28.0 (22.0)	28.7 (28.7)	26.2 (22.3)	.48
**Sheltering status**					
Unsheltered	265	42 (15.9)	27 (14.8)	15 (18.1)	.06
Emergency sheltered	265	46 (17.4)	39 (21.4)	7 (8.4)	
Transitional housing	265	97 (36.6)	61 (33.5)	36 (43.4)	
Doubled-up	265	80 (30.2)	55 (30.2)	25 (30.1)	
**Health**					
Overall health status,^f^ mean (SD)	261	2.5 (0.8)	2.5 (0.8)	2.5 (0.8)	.90
Received acute care services during 12 consecutive months of enrollment	264	190 (72.0)	125 (68.7)	65 (79.3)	.08
**Medical condition**					
Any medical problem	265	227 (85.7)	155 (85.2)	72 (86.8)	.73
Arthritis/chronic pain	259	111 (42.9)	78 (44.3)	33 (39.8)	.49
Hypertension	263	87 (33.1)	56 (31.1)	31 (37.4)	.32
Hepatitis/cirrhosis	261	66 (25.3)	40 (22.4)	26 (31.7)	.11
Emphysema/asthma/COPD	262	64 (24.4)	46 (25.4)	18 (22.2)	.58
Gastrointestinal disorders	263	52 (19.8)	35 (19.4)	17 (20.5)	.84
Heart disease	263	26 (9.9)	15 (8.3)	11 (13.3)	.21
Seizure disorder	262	19 (7.3)	13 (7.2)	6 (7.3)	>.99
Cancer	261	15 (5.8)	12 (6.7)	3 (3.7)	.40
**Mental health condition**					
Any mental health condition	265	207 (78.1)	138 (75.8)	69 (83.1)	.18
Depression	260	180 (69.2)	122 (67.4)	58 (73.4)	.33
Anxiety	261	165 (63.2)	111 (61.7)	54 (66.7)	.44
Posttraumatic stress disorder	246	125 (50.8)	85 (50.9)	40 (50.6)	.97
Bipolar disorder	245	47 (19.2)	33 (19.6)	14 (18.2)	.79
**Substance use or abuse**					
Any drinking in past 6 months	265	162 (61.1)	114 (62.6)	48 (57.8)	.46
Cocaine use in past 6 months	265	60 (22.6)	44 (24.2)	16 (19.3)	.37
Heroin or nonprescribed opiate use in past 6 months	266	24 (9.0)	19 (10.4)	5 (6.0)	.25
**Injured in altercation in past 6 months**	264	42 (15.9)	35 (19.3)	7 (8.4)	.03

Abbreviations: COPD, chronic obstructive pulmonary disease; H-PACT, Homeless-Patient Aligned Care Team; PACT, Patient Aligned Care Team; SD, standard deviation.

a Built on the framework of PACT, H-PACT addresses issues of access, treatment engagement, competing priorities, and the social determinants of health that are associated with homelessness.

b Percentages are based on number of respondents who answered question.

c PACT is a primary care–based model constructed on the principles of patient centeredness, interdisciplinary teamwork, efficiency, comprehensive whole person–oriented longitudinal care, and active communication and coordination (24).

d Determined by *t* test (difference in means) and χ^2^ analyses (difference in frequencies).

e The median (interquartile range) was 24 (7–48) for H-PACT, 18 (6–48) for PACT, and 24 (7–48) overall.

f Self-rated Likert scale from 1 (excellent) to 5 (poor).

Almost 86% overall reported at least one medical condition, and 78.1% reported at least one mental health condition. The most common medical conditions were arthritis/chronic pain (42.9%), hypertension (33.1%), hepatitis/cirrhosis (25.3%), and emphysema/asthma/chronic obstructive pulmonary disease (24.4%). The most common mental health conditions were depression (69.2%), anxiety (63.2%), and posttraumatic stress disorder (50.8%). The majority in both groups reported using alcohol in the past 6 months (61.1%); 22.6% used cocaine, and 9.0% used opiates (including heroin and nonprescribed opiates). A significantly greater percentage in the H-PACT group had been injured in an altercation in the previous 6 months (19.3% vs 8.4%). (Table 1).

At study completion, we found no difference between groups in their satisfaction with care received at the VA, and ratings were favorable in both groups (Table 2).

**Table 2:** Satisfaction With Care in the US Department of Veterans Affairs’ PACT or H-PACT, Study on Population-Tailored Care for Homeless Veterans and Acute Care Use, Cost, and Satisfaction, June 2012–January 2014^a^

Statement	Mean (Standard Deviation)		*P* Value^d^
	H-PACT^b^ (N = 183)	PACT^c^ (N = 83)	
Staff are respectful	1.5 (0.7)	1.4 (0.6)	.66
Staff are sensitive to needs	1.6 (0.9)	1.6 (0.8)	.84
Staff not as competent and staff in non-VA care	4.3 (1.1)	4.0 (1.2)	.07
Care is helpful	1.3 (0.7)	1.4 (0.9)	.20
Care is better than elsewhere	1.4 (0.8)	1.6 (0.9)	.36
Long wait	3.6 (1.3)	3.4 (1.3)	.31
More affordable than non-VA care	1.2 (0.7)	1.1 (0.4)	.54
All questions answered	1.6 (1.0)	1.8 (1.0)	.36
Included in care decisions	1.6 (1.0)	1.7 (1.0)	.85
Provider listens to you	1.5 (0.9)	1.6 (1.0)	.31
Get everything you need without being sent elsewhere	1.8 (1.1)	2.0 (1.2)	.26
Treated better because homeless	3.5 (1.6)	3.6 (1.4)	.66
Treated worse because homeless	4.2 (1.3)	4.3 (1.2)	.65
Hard time getting there	3.6 (1.5)	3.8 (1.4)	.44
Too much bureaucracy	3.5 (1.5)	3.3 (1.5)	.34

Abbreviations: H-PACT, Homeless-Patient Aligned Care Team; PACT, Patient Aligned Care Team; SD, standard deviation; VA, US Department of Veterans Affairs.

a Perceptions of care received at VA, self-rated on a Likert scale of 1 (strongly agree) to 5 (strongly disagree). Patients were enrolled during June 2012 through January 2014; data on health care use were collected for 12 consecutive months for each participant enrolled; at the end of the 12 months, each participant completed a survey on program satisfaction.

b Built on the framework of PACT, H-PACT addresses issues of access, treatment engagement, competing priorities, and the social determinants of health that are associated with homelessness.

c PACT is a primary care–based model constructed on the principles of patient centeredness, interdisciplinary teamwork, efficiency, comprehensive whole person–oriented longitudinal care, and active communication and coordination (24).

d Determined by *t* test.

During the study period, veterans in the H-PACT and PACT groups had similar numbers of primary care visits (H-PACT, 8.8 visits; PACT, 7.1 visits), specialty care visits (H-PACT, 3.1 visits; PACT, 3.6 visits), ED visits (H-PACT, 2.6 visits; PACT, 2.9 visits), and any hospitalizations (H-PACT, 0.4 hospitalizations; PACT, 0.6 hospitalizations) (Table 3). Veterans in the PACT group had significantly more mental health care visits (13.4 vs 8.8 visits) and prescriptions filled (58.8 vs 40.5 fills), whereas veterans in the H-PACT group had more PCP-specific visits (5.1 vs 3.6 visits) and social work visits (4.6 vs 2.7 visits) and fewer ED visits for ambulatory-care–sensitive conditions (0 vs 0.2 visits). Overall costs were significantly higher for PACT-based care ($37,415 vs $28,036), with only primary care costs (higher in H-PACTs) and mental health/substance abuse treatment costs (higher in PACTs) significantly different.

**Table 3:** Health Services Use and Costs of US Department of Veterans Affairs’ H-PACT and PACT, Study on Population-Tailored Care for Homeless Veterans and Acute Care Use, Cost, and Satisfaction, June 2012–January 2014

Variable	H-PACT^a^ (N = 183)	PACT^b^ (N = 83)	*P* Value^c^
**No. of visits, mean (SD)**			
Primary care provider and nursing visits	8.8 (7.1)	7.1 (6.4)	.06
Primary care provider-specific visits	5.1 (4.1)	3.6 (2.8)	.001
Specialty care visits	3.1 (5.0)	3.6 (4.5)	.41
Social work visits	4.6 (3.7)	2.7 (2.1)	.001
Mental health care visits	8.8 (11.8)	13.4 (14.3)	.01
All emergency department visits	2.6 (4.4)	2.9 (3.9)	.57
Emergency department visits for ambulatory-care–sensitive conditions	0 (0.2)	0.2 (0.6)	.04
**No. of hospitalizations, mean (SD)**			
Hospitalizations (acute medical/surgical, mental health, and substance abuse)	0.4 (0.8)	0.6 (1.2)	.06
Hospitalizations not at a VA Hospital	0 (0.1)	0.1 (9.7)	.29
**30-Day prescription drug fills, mean (SD)**	40.5 (39.5)	58.8 (53.9)	.001
**No. (%) of participants accessing . . .**			
Psychiatry	102 (56.0)	52 (63.4)	.26
Psychology	59 (32.4)	32 (39.0)	.30
Group therapy	73 (40.1)	44 (53.7)	.04
Emergency department (any)	111 (61.0)	54 (65.9)	.45
Emergency department (mental health–related)	62 (34.1)	39 (47.6)	.04
Hospitalization	42 (23.1)	29 (35.4)	.04
**Costs, mean (SD), $**			
Overall	28,036 (27,036)	37,415 (36,872)	.04
Primary care	2,947 (2,511)	2,266 (2,266)	.03
Specialty care	1,824 (3,838)	1,880 (3,131)	.90
Mental health-substance abuse treatment	3,378 (4,759)	4,770 (5,084)	.03
Emergency department (all)	1,978 (3,627)	2,235 (4,076)	.61
Emergency department for ambulatory care–sensitive conditions	19 (165)	105 (517)	.14
Non-VA based care	19 (252)	1,035 (8,298)	.27
Hospitalization	5,530 (18,138)	10,429 (24,427)	.10
Prescription drugs	1,698 (2,441)	3,181 (11,483)	.25

Abbreviations: H-PACT, Homeless-Patient Aligned Care Team; PACT, Patient Aligned Care Team; SD, standard deviation; VA, US Department of Veterans Affairs.

a Built on the framework of PACT, H-PACT addresses issues of access, treatment engagement, competing priorities, and the social determinants of health that are associated with homelessness.

b PACT is a primary care–based model constructed on the principles of patient centeredness, interdisciplinary teamwork, efficiency, comprehensive whole-person–oriented longitudinal care, and active communication and coordination (24).

c Determined by *t* test (difference in means) and χ^2^ analyses (difference in frequencies).

A significantly greater percentage of veterans in the PACT arm were hospitalized for any cause (35.4% vs 23.1%), had a mental health–related ED visit (47.6% vs 34.1%), or attended group therapy (53.7% vs 40.1%). We identified 2 independent predictors of acute care use in the multivariate logistic regression: assignment to the H-PACT group protected against having an acute care event (odds ratio [OR] = 0.41; 95% confidence interval [CI], 0.21–0.80) as did self-reporting good or better overall health (OR = 0.63; 95% CI, 0.42–0.95). Higher numbers of visits to the primary care team (OR = 1.10; 95% CI, 1.01–1.19) and higher number of psychiatrist visits were associated with increased odds of having an acute care event (OR = 2.05; 95% CI, 1.15–3.65).

## Discussion

Homeless patients enrolled in both care models demonstrated high levels of treatment engagement in primary care, specialty care, and mental health care services, which reflects well on both care models and their accessibility to vulnerable and disadvantaged veteran populations. Furthermore, homeless veterans enrolled in both models appeared to be satisfied with the care they received. However, the cost of caring for this population was significantly lower in the H-PACT group, with fewer patients needing hospitalization, visiting an ED for a mental health need, or having ED-based care for ambulatory-care–sensitive conditions. In the logistic regression model, patients enrolled in H-PACT were less than half as likely to use acute care services. In contrast, higher rates of both primary care and psychiatrist visits were independently associated with increased acute care use. We suspect the independent effect of H-PACT enrollment on acute care use reflects the enhanced capacity of the population-tailored model to affect several social drivers of acute care use. An analysis of the national H-PACT program found that sites providing more on-site support (meals and food assistance, clothing, transportation, showers, and hygiene kits), sites with enhanced access accommodations for on-demand care, and sites with greater integration of housing interventions all had lower levels of ED and inpatient care use (28).

Although we found no difference in the overall number of hospitalizations and ED visits between groups, the proportion of patients in the H-PACT group who were hospitalized or accessed the ED for mental health care needs was substantially lower. We suspect the observed non-difference may reflect the relatively low incident rate for these acute care events among the veterans in our study, which was well-engaged in an ambulatory model. However, the lower proportion of H-PACT patients being hospitalized also suggests higher efficacy in this care model. This difference was as robust as noted in previous studies (19–22), which may be in part due to the high level of care and treatment engagement in the PACT group.

The economic implications of our findings are considerable for the health planning needs of accountable care organizations and other groups assuming capitated risk for high-cost groups like homeless persons. The cost of providing care to the matched cohort of homeless veterans in the H-PACT model was $9,379 per patient per year less than in the PACT model. Although primary care–related costs were higher in the H-PACT group, costs in all other categories were lower, and most of the cost savings came from both VA and non-VA hospitalizations. We recognize that these cost savings may not necessarily be realized in integrated systems such as the VHA, where many unit costs (eg, staffing) are relatively fixed; however, third-party payer costs and capitated savings are potentially large and reflect both clinical and social benefit. These findings also underscore the potential impact that VA care can have on non-VA community health systems and the importance of care coordination.

Our findings also highlight the importance of considering care delivery strategies within a population health framework that goes beyond disease-based stratification of high-cost patients. Much of population health modeling is organized according to diseases such as diabetes or congestive heart failure (33). Although these approaches concentrate and coordinate complex treatment regimens for patients with substantial morbidity, they do not necessarily address some of the access and treatment engagement barriers driven by lack of trust, perceived stigma, past experiences (15,34), or competing sustenance needs (35) encountered by homeless and other socially disadvantaged populations.

Our study has several limitations. Although the quasi-experimental study design is an improvement over the design of previous studies, unmeasured differences between the groups might have been controlled for in a randomized controlled trial study design, which was not chosen for reasons noted. The study population was limited to predominantly male homeless veterans and may not be generalizable to other population groups such as nonveterans, women, and younger persons, who may have different obstacles to access, treatment engagement, or drivers of poor health outcomes. Basing the study in VHA and using electronic medical records allowed us to more efficiently capture data on care use across the continuum of services and events for the entire sample, although we may not have captured all data on care received in other health systems. Recording of non-VA care events was subject to recall bias. Further research is needed to determine whether our findings are reproducible. Finally, our study was conducted in urban centers on the East and West coasts and may not be generalizable to rural communities or settings with fewer homeless persons, where adequate economies of scale may not exist to concentrate care and services in this type of model.

Our study suggests that a population-tailored medical home approach for socially disadvantaged populations can both reduce reliance on acute care service use and generate significant cost savings. Our findings have implications for health systems managers and policy planners who are considering how to optimize capitated care for these population groups in VHA, Medicaid-managed care plans, or accountable care organizations.

## References

[1] Institutes of Medicine (US) Committee on Health Care for Homeless People. Homelessness, health, and human needs. Washington (DC): National Academies Press; 1988.

[2] Adams J , Rosenheck R , Gee L , Seibyl CL , Kushel M . Hospitalized younger: a comparison of a national sample of homeless and housed inpatient veterans. J Health Care Poor Underserved 2007;18(1):173–84. 10.1353/hpu.2007.0000 17337806

[3] Argintaru N , Chambers C , Gogosis E , Farrell S , Palepu A , Klodawsky F , A cross-sectional observational study of unmet health needs among homeless and vulnerably housed adults in three Canadian cities. BMC Public Health 2013;13(1):577. 10.1186/1471-2458-13-577 23764199 PMC3691921

[4] Baggett TP , O’Connell JJ , Singer DE , Rigotti NA . The unmet health care needs of homeless adults: a national study. Am J Public Health 2010;100(7):1326–33. 10.2105/AJPH.2009.180109 20466953 PMC2882397

[5] Hastings SN , Smith VA , Weinberger M , Schmader KE , Olsen MK , Oddone EZ . Emergency department visits in Veterans Affairs medical facilities. Am J Manag Care 2011;17(6 Spec No):e215–23. 21756015 PMC6519060

[6] Chambers C , Chiu S , Katic M , Kiss A , Redelmeier DA , Levinson W , High utilizers of emergency health services in a population-based cohort of homeless adults. Am J Public Health 2013;103(Suppl 2):S302–10. 10.2105/AJPH.2013.301397 24148033 PMC3969147

[7] Baggett TP , Hwang SW , O’Connell JJ , Porneala BC , Stringfellow EJ , Orav EJ , Mortality among homeless adults in Boston: shifts in causes of death over a 15-year period. JAMA Intern Med 2013;173(3):189–95. 10.1001/jamainternmed.2013.1604 23318302 PMC3713619

[8] Alexander JA , Bae D . Does the patient-centred medical home work? A critical synthesis of research on patient-centred medical homes and patient-related outcomes. Health Serv Manage Res 2012;25(2):51–9. 10.1258/hsmr.2012.012001 22673694

[9] Maeng DD , Graf TR , Davis DE , Tomcavage J , Bloom FJ Jr . Can a patient-centered medical home lead to better patient outcomes? The quality implications of Geisinger’s ProvenHealth Navigator. Am J Med Qual 2012;27(3):210–6. 10.1177/1062860611417421 21852292

[10] O’Neill JL , Cunningham TL , Wiitala WL , Bartley EP . Collaborative hypertension case management by registered nurses and clinical pharmacy specialists within the Patient Aligned Care Teams (PACT) model. J Gen Intern Med 2014;29(Suppl 2):S675–81. 10.1007/s11606-014-2774-4 24715403 PMC4070225

[11] Flottemesch TJ , Anderson LH , Solberg LI , Fontaine P , Asche SE . Patient-centered medical home cost reductions limited to complex patients. Am J Manag Care 2012;18(11):677–86. 23198711

[12] McMurray-Avila M . Organizing health services for homeless people: a practical guide. Nashville (TN): National Health Care for the Homeless Council; 1997.

[13] Reynolds HY . Free medical clinics: helping indigent patients and dealing with emerging health care needs. Acad Med 2009;84(10):1434–9. 10.1097/ACM.0b013e3181b6c3eb 19881438

[14] Kertesz SG , Holt CL , Steward JL , Jones RN , Roth DL , Stringfellow E , Comparing homeless persons’ care experiences in tailored versus nontailored primary care programs. Am J Public Health 2013;103(Suppl 2):S331–9. 10.2105/AJPH.2013.301481 24148052 PMC3969114

[15] Wen CK , Hudak PL , Hwang SW . Homeless people’s perceptions of welcomeness and unwelcomeness in healthcare encounters. J Gen Intern Med 2007;22(7):1011–7. 10.1007/s11606-007-0183-7 17415619 PMC2219712

[16] O’Toole TP , Bourgault C , Johnson EE , Redihan SG , Borgia M , Aiello R , New to care: demands on a health system when homeless veterans are enrolled in a medical home model. Am J Public Health 2013;103(Suppl 2):S374–9. 10.2105/AJPH.2013.301632 24148042 PMC3969111

[17] Chambers C , Katic M , Chiu S , Redelmeier DA , Levinson W , Kiss A , Predictors of medical or surgical and psychiatric hospitalizations among a population-based cohort of homeless adults. Am J Public Health 2013;103(Suppl 2):S380–8. 10.2105/AJPH.2013.301646 24148040 PMC3969145

[18] Saab D , Nisenbaum R , Dhalla I , Hwang SW . Hospital readmissions in a community-based sample of homeless adults: a matched-cohort study. J Gen Intern Med 2016;31(9):1011–8. 10.1007/s11606-016-3680-8 27197973 PMC4978672

[19] Han B , Wells BL . Inappropriate emergency department visits and use of the Health Care for the Homeless Program services by homeless adults in the northeastern United States. J Public Health Manag Pract 2003;9(6):530–7. 10.1097/00124784-200311000-00014 14606193

[20] Blue-Howells J , McGuire J , Nakashima J . Co-location of health care services for homeless veterans: a case study of innovation in program implementation. Soc Work Health Care 2008;47(3):219–31. 10.1080/00981380801985341 19042482

[21] O’Toole TP , Buckel L , Bourgault C , Blumen J , Redihan SG , Jiang L , Applying the chronic care model to homeless veterans: effect of a population approach to primary care on utilization and clinical outcomes. Am J Public Health 2010;100(12):2493–9. 10.2105/AJPH.2009.179416 20966377 PMC2978184

[22] McGuire J , Gelberg L , Blue-Howells J , Rosenheck RA . Access to primary care for homeless veterans with serious mental illness or substance abuse: a follow-up evaluation of co-located primary care and homeless social services. Adm Policy Ment Health 2009;36(4):255–64. 10.1007/s10488-009-0210-6 19280333

[23] Stewart B. McKinney Homeless Assistance Act. Public Law 100-77. 42 USC 11411 and 11412.

[24] Rosland AM , Nelson K , Sun H , Dolan ED , Maynard C , Bryson C , The patient-centered medical home in the Veterans Health Administration. Am J Manag Care 2013;19(7):e263–72. 23919446

[25] Nelson KM , Helfrich C , Sun H , Hebert PL , Liu CF , Dolan E , Implementation of the patient-centered medical home in the Veterans Health Administration: associations with patient satisfaction, quality of care, staff burnout, and hospital and emergency department use. JAMA Intern Med 2014;174(8):1350–8. 10.1001/jamainternmed.2014.2488 25055197

[26] Piette JD , Holtz B , Beard AJ , Blaum C , Greenstone CL , Krein SL , ; Ann Arbor PACT Steering Committee. Improving chronic illness care for veterans within the framework of the Patient-Centered Medical Home: experiences from the Ann Arbor Patient-Aligned Care Team Laboratory. Transl Behav Med 2011;1(4):615–23. 10.1007/s13142-011-0065-8 24073085 PMC3717663

[27] Randall I , Mohr DC , Maynard C . VHA patient-centered medical home associated with lower rate of hospitalizations and specialty care among veterans with posttraumatic stress disorder. J Healthc Qual 2015. 10.1111/jhq.12092 28481843

[28] O’Toole TP , Johnson EE , Aiello R , Kane V , Pape L . Tailoring care to vulnerable populations by incorporating social determinants of health: The Veterans Health Administration’s “Homeless Patient Aligned Care Team” Program. Prev Chronic Dis 2016;13:E44. 27032987 10.5888/pcd13.150567 PMC4825747

[29] American College of Physicians. Practice resources. https://www.acponline.org/practice-resources. Accessed September 19, 2017.

[30] Sherbourne CD , Stewart AL . The MOS social support survey. Soc Sci Med 1991;32(6):705–14. 10.1016/0277-9536(91)90150-B 2035047

[31] Stanford University. Stanford University’s chronic disease self-management program: curriculum and evidence. http://patienteducation.stanford.edu/research/healthdistress.html. Accessed on October 16, 2008.

[32] US Department of Health and Human Services, Agency for Healthcare Research and Quality. Guide to prevention quality indicators: hospital admission for ambulatory care sensitive conditions. AHRQ publication no. 02-R0203. https://www.ahrq.gov/downloads/pub/ahrqqi/pqiguide.pdf. Accessed on September 18, 2015.

[33] Smith JJ , Johnston JM , Hiratsuka VY , Dillard DA , Tierney S , Driscoll DL . Medical home implementation and trends in diabetes quality measures for AN/AI primary care patients. Prim Care Diabetes 2015;9(2):120–6. 10.1016/j.pcd.2014.06.005 25095763

[34] O’Toole TP , Johnson EE , Redihan S , Borgia M , Rose J . Needing primary care but not getting it: the role of trust, stigma and organizational obstacles reported by homeless veterans. J Health Care Poor Underserved 2015;26(3):1019–31. 10.1353/hpu.2015.0077 26320930

[35] Gelberg L , Gallagher TC , Andersen RM , Koegel P . Competing priorities as a barrier to medical care among homeless adults in Los Angeles. Am J Public Health 1997;87(2):217–20. 10.2105/AJPH.87.2.217 9103100 PMC1380797

